# Health-related quality of life in Danish cancer survivors referred to a late effects clinic

**DOI:** 10.2340/1651-226X.2024.39937

**Published:** 2024-06-16

**Authors:** Lærke Kjær Tolstrup, Karin B. Dieperink, Marieke van Leeuwen, Sören Möller, Linnea Fechner, Line Helene Clausen, Thea Otto Mattsson

**Affiliations:** a Department of Oncology, Odense University Hospital, Odense C, Denmark; b Department of Clinical Research, University of Southern Denmark, Odense Denmark; c Division of Psychosocial Research & Epidemiology, the Netherlands Cancer Institute, Amsterdam, the Netherlands; d Open Patient data Explorative Network, Odense University Hospital, Odense C, Denmark

**Keywords:** Cancer, late effects, late effects clinic, health-related quality of life, survivorship, PRO, EORTC QLQ-SURV100

## Abstract

**Purpose:**

The Region of Southern Denmark has recently established four late effects clinics to help cancer survivors suffering from complex and severe late effects. This study aimed to capture and analyze the full range of physical, mental, and psychosocial issues using patient-reported outcomes. Moreover, we aimed to describe demographic data and the type and severity of the late effects.

**Methods:**

A prospective cohort study was conducted among cancer survivors referred to a late effects clinic. Before their first appointment, patients completed the European Organization for Research and Treatment of Cancer Quality of Life cancer survivorship core questionnaire (EORTC QLQ-SURV100). We compared mean scores of the EORTC QLQ-SURV100 scales that were comparable to the scales/items from the EORTC QLQ-C30 questionnaire with norm data for the Danish population and EORTC reference values.

**Results:**

All patients referred to the clinic within its first 2 years were included (*n* = 247). The mean age was 57 [23–85] years and 74% were females. The most common cancer diagnoses was breast cancer (39%). The five most commonly reported late effects were fatigue (66%), pain (51%), cognitive impairment (53%), sleep problems (42%), and neuropathy (40%). A total of 236 of the patients entering the clinic completed QLQ-SURV100. They reported significantly worse mean scores on all scales compared to the Danish norm population and EORTC reference values for pretreatment cancer patients, *p* < 0.001. Effect sizes were moderate or large for all scales.

**Interpretation:**

In this study, we collected demographic data and described the late effects presented by the patents referred to the clinic. Moreover, we captured and analyzed the full range of physical, mental, and psychosocial issues using QLQ-SURV100. Patients referred to the Late Effects Clinic (LEC) had a number of late effects and reported a significantly lower health-related quality of life compared to the general Danish population and patients who have just been diagnosed with cancer, suggesting the aim of helping patients suffering from late effects gain a better quality of life is in dire need.

## Introduction

The number of people diagnosed with cancer is increasing. A majority of them become long-term survivors due to early cancer detection and improved treatment modalities [1]. By 2030, the number of cancer survivors worldwide is projected to increase to 22.1 million [2]. In Denmark at the end of 2021, approximately 6% of the population was living with a cancer diagnosis [3].

According to the Danish Health Authority [4], at least 50% of cancer survivors experience one or more late effects originating from cancer and/or its treatment. In a Danish context, late effects are defined as health problems that occur during cancer treatment and become chronic or develop months or years after treatment has ended [5]. This includes physical, psychological, emotional, social, and spiritual symptoms or new primary cancers. A review from 2020 describes that three out of four cancer survivors develop late effects [2]. These late effects may interfere negatively with the patient’s everyday life and affect their health-related quality of life (HRQoL) [6]. A meta-analysis from 2020 concluded that HRQoL among cancer survivors was significantly impacted 2 or more years after diagnosis [7].

In recent years, there has been an increasing awareness of mitigating the negative impact of long-term effects that patients may experience after their cancer trajectory. Jefford et al. suggest making a shift in the current models of care away from the predominant focus on the detection of recurrence toward improving other aspects of care, including patients’ Quality of life (QoL), functional outcomes, and risk of recurrence [8]. A review by Emery et al. is in line with this and suggests an approach using novel therapeutic, behavioral, and healthcare system interventions to remedy long-term effects such as pain, fatigue, neuropathy, and fear of recurrence [1].

Despite these newly developed recommendations, many cancer survivors describe current practice at their oncological follow-up visits as primarily focusing on the efficacy of their treatment and detection of relapse and less on addressing potential late effects caused by their treatment [6]. Studies show that psychosocial and existential challenges are not adequately addressed in the existing healthcare system [8–10]. Cancer survivors report that this is particularly true for problems that physicians might consider medically nonthreatening or for which no effective medical treatment is known [11].

Similarly, late effects can place a burden on family caregivers or other informal caregivers, as cancer survivors may continue to need support during the various potential trajectories associated with long-term survivorship. It can pose a challenge for family caregivers to manage both their own and the cancer survivors’ concerns that cancer might return or progress, thereby complicating readjustment to life after cancer [12]. In addition, the costs to society as a whole are affected due to a lack of productivity in cancer survivors as well as the family caregivers [9, 13] by, for example, reduced ability to work [14].

In accordance with the unmet needs and suggested shift in perspective, The Region of Southern Denmark decided in April 2021 to establish a ‘Single entrance for patients with complex late effects after cancer and cancer treatment’. This initiative resulted in the development of four regional late effects clinics with the shared goal of helping cancer survivors suffering from late effects of cancer trajectory and treatment. The regional clinics are placed within the existing departments of oncology at four regional hospitals, aiming to ensure equal access for all patients.

As described, late effects are poorly identified and addressed within the current models of care [8]. Moreover, there is a lack of knowledge about the exact extent of late effects after cancer treatment as well as their impact on patients’ QoL. The newly established Late Effects Clinics (LECs) provide a novel opportunity to examine both the number and severity/complexity of *unmet* needs concerning the late effects experienced by a subset of cancer survivors within a real-life clinical setting.

This study aimed to capture and analyze the full range of physical, mental, and psychosocial issues among Danish cancer survivors at the time of their referral (baseline) to a Danish LEC using patient-reported outcomes. Furthermore, we describe demographic data and the type and impact on QoL of the late effects experienced by these patients.

## Material and methods

### Setting and patients

A cohort study was conducted with the inclusion of all patients referred to a novel LEC for patients experiencing complex late effects after cancer and its treatment. Late effects were considered complex if they could not be managed by clinicians in the traditional healthcare system. Often, the patients had several late effects that occured in symptom clusters. The clinic is located at the Department of Oncology, Odense University Hospital within the Region of Southern Denmark, and opened in February 2022. The criteria for referral were complex late effects following cancer and its treatment and covering all cancer types in adult cancers. Referrals were initiated on behalf of the patients’ general practitioner, surgeon, oncologist, hematologist, or any healthcare professional within the Department of Oncology at Odense University Hospital. The patients had to live inside the hospital catchment area on the Island of Funen and the South Funen Archipelago. The clinic exclusively accepted patients with no current evidence of active cancer or cancer recurrence. To ensure a high-quality presentation of our findings, the STROBE guidelines were followed [15].

### Patient flow

Following referral, the patients were booked for a consultation with two different healthcare professionals from the clinic, that is physician, nurse, psychologist, or sexologist depending on the type of late effects described in the referral. As recommended by current evidence [1], the first consultation focused on reviewing the patients’ history of cancer and past cancer treatments; the patients and their family caregivers were encouraged to share information regarding both physical and psychosocial late effects as well as any other issues affecting their daily life. The following consultations were held in accordance with the individual treatment plans of the patients, developed through multidisciplinary team conferences, and shared decisions with the patients. Interventions were multidisciplinary, individual, or group based and were conducted, when relevant, in collaboration with other hospital specialties and through municipality rehabilitation programs.

### Database

The patients’ data were captured electronically in a REDCap database upon the patients’ consent. We collected information on age, gender, and demographic data such as education, employment, living with a partner or alone, and number of children. In addition, we collected disease characteristics, that is diagnoses, cancer treatment, type, and number of late effects. The late effects captured in the database were collected during the first consultation where the patients´ circumstances were thoroughly examined. Accordingly, the two clinicians who met the patients at the first encounter decided which late effects to report in the database based on medical records, patient-reported outcomes, and conversation with the patient.

### QLQ-SURV100

Before their first appointment at the clinic, patients were asked to sign an informed consent form and fill out the European Organization for Research and Treatment of Cancer Quality of Life cancer survivorship core questionnaire (EORTC QLQ-SURV 100). This questionnaire [16] has been developed to assess HRQoL in disease-free cancer survivors. The questionnaire aims to capture short- and long-term adverse physical and psychosocial effects as well as socioeconomic challenges, including the positive psychosocial effects that might impact QoL in the survivorship context. Hence capturing the full range of issues relevant to the disease-free cancer survivors [16]. The QLQ-SURV100 consists of 100 items, including a global health status/QoL scale, functional scales, symptom scales, one symptom checklist, and single items. The scoring approach is similar to that of the QLQ-C30; all scales and single items score from 0 to 100. A high score for a functional scale and the global health status represents a high level of functioning/global health status, whereas a high score for a symptoms scale represents a high level of symptomatology. The scales from the QLQ-SURV100 cannot be compared directly with the scales from QLQ-C30 as items that were no longer applicable in disease-free cancer survivors were excluded, while items specific to disease-free cancer survivors were added. However, the scoring manual for SURV100 describes which items from the QLQ-SURV100 can be used to calculate the equivalent QLQ-C30 scales for Global health, Physical functioning, Role functioning, Emotional functioning, Social interference, Fatigue, Pain, Sleep problems, and Financial difficulties. This makes it possible to compare our findings with previous data published on QLQ-30. In addition, we also report and compare data on the new scales from SURV100.

The EORTC QLQ-SURV100 questionnaire served as a screening and dialog tool at the first consultation in the clinic, helping the patients to reflect on their situation and enabling the healthcare professionals to address all areas of late effects developed by the patients’ cancer treatment. An ongoing investigation is elucidating how patients and clinicians experience the use of the SURV100 questionnaire through focus groups with clinicians and individual inteviews with patients. In addition to completing the questionnaire at baseline, the patients were asked to complete the questionnaire at the time of their last consultation in the LEC and again after six, 12, and 24 months to investigate whether the interventions had been helpful and improved the patients’ HRQoL both short and long term. In this paper, we present baseline data and compare them to the Danish norm population [17] and EORTC reference values for pretreatment cancer patients [18].

### Statistical considerations

We used descriptive statistics to describe demographic data and the type of late effects. We compared the mean scores of the EORTC QLQ-SURV100 scales and the calculated equivalent QLQ-C30 scales with norm data for the Danish population. The norm data had been collected by the EORTC for the purposes of comparing cancer patient data with data from the general population for individual countries in Europe and North America [17]. Furthermore, we compared our data with data from the manual on EORTC reference values, which consists of data from various EORTC studies. The manual is based on baseline data. Thus, the patients had all been diagnosed with cancer but were not receiving treatment yet [18]. Data were compared using a one-sample z-test. Furthermore, we used Cohen’s d to compare means between the groups to determine whether a potential observed effect was meaningful and had practical significance [19]. Stata 18 was used to analyze data [20].

## Results

### Database

All patients referred to the clinic within its first 2 years were included (*n* = 247) in the database. The mean age was 57 [range 23–85] years, and 74% were females. The most common cancer diagnoses were breast (39%), colorectal (14%), gynecological (12%), and head and neck (7%) cancer. Twenty-eight per cent of the patients lived alone. Forty-one per cent of the patients were referred to the LEC by their general practitioner, and 46% were referred by a clinician within the Department of Oncology at Odense University Hospital (Table 1). The five most commonly reported late effects were fatigue (66%), pain (51%), cognitive impairment (53%), sleep problems (42%), and neuropathy (40%). Ninety per cent of the patients reported experiencing more than one late effect, and 59% experienced 4 or more late effects. Patients experienced from one to nine late effects with a mean of five.

**Table 1:** Characteristics of 247 cancer survivors at first visit at a late effects clinic (LEC).

	Total population
	*n* (%)
Sociodemographic variables Gender, *n* (%)	
Female	183 (74)
Male	64 (26)
Age at first visit, mean (SD)	57 (12.5)
Living with partner, *n* (%)	177 (72)
Children <18 years, *n* (%)	68 (28)
Educational attainment, *n* (%)	
Primary education	18 (7)
Upper secondary/vocational education	79 (33)
Higher (vocational)/university education	116 (47)
Other	31 (13)
Referrals, *n* (%)	
General practitioner	102 (41)
Department of Oncology	113 (46)
Other	32 (13)
Cancer diagnosis	
Breast	96 (39)
Digestive system including pancreatic	35 (14)
Lung	11 (5)
Genital (male)	15 (6)
Genital (female)	29 (12)
Head and neck	18 (7)
Hematological	26 (10)
Melanoma	10 (4)
Other	7 (3)
Age at diagnosis, mean (SD)	51 (13.2)
Years since primary diagnosis, mean (SD)	5.1 (6.4)
Treatment modalities^1^, *n* (%)	
Surgery	196 (80)
Radiotherapy	136 (55)
Chemotherapy	187 (76)
Immunotherapy	18 (7)
Targeted treatment	7 (3)
Endocrine treatment	63 (26)
Other^2^	36 (15)
Years since the last treatment, mean (SD)	3.1 (4.6)
Comorbidity, *n* (%)	
Yes	157 (64)
No	81 (33)
Unknown	9 (3)

LEC: Late Effects Clinics; N: number; SD: standard deviation.

1 Categories are not mutually exclusive.

2 Other include zoledronic acid, radioactive iodine, bone marrow, and stem cell transplantat.

### Patient-reported outcomes

Of the 247 patients included in this study, 236 completed the QLQ-SURV100 before their first consultation. Eleven patients did not complete the questionnaire due to cognitive, logistic, or language challenges. Unpublished data from focus groups with clinicians and individual interviews with patients demonstrate that clinicians found the questionnaire useful for screening, ensuring that all problems were addressed in the initial consultation. Similarly, the majority of patients believed that the questions were relevant, and the questionnaire was not too burdensome to complete despite the high number of questions, which is supported by the high response rate. The patients entering our clinic reported significantly lower mean scores on all functional scales and higher scores on all symptom scales both regarding the calculated C30-scales and the SURV100 scales compared to the Danish norm population and EORTC reference values for pretreatment cancer patients, *p* < 0.001 (Table 2). Comparing our data with Norm data (Table 2), effect sizes were considered either large (*d* ≥ 0.8) for five scales or moderate (*d* = 0.5) for five scales indicating that the observed differences were for the most part substantial and had practical significance. When it comes to comparing our data with EORTC reference data (Table 2), effect sizes were considered large for two scales and moderate for eight scales, suggesting at least a noticeable difference with practical implications.

**Table 2:** Overview of the scales from the EORTC SURV100 and the EORTC QLQ-C30 scales questionnaires and comparisons with norm data and EORTC reference values for selected scales, including effects sizes.

QOL-SURV100 scales and corresponding QOL-C30 scales (*n*)	QOL-SURV100Danish data LEC baseline Mean (SD)	Scales of the QLQ-C30 calculated with items from QLQ-SURV100 LEC baseline Mean (SD)	Norm data QLQ-C30 General population (Denmark)^1^ Mean (SD)	Effect size estimation between Norm data and SURV100/C30 Cohen’s d	EORTC QLQ-C30 Reference Values Manual^2^ Mean	Effect size estimation between Reference Values and SURV100/C30 Cohens’d
Global health score	51.8 (18.4)	51.8 (18.4)	67.0 (23.4)	0.7/0.7	61.3 (24.2)	0.4/0.4
Physical functioning	63.2 (24.7)	63.2 (24.7)	84.2 (20.4)	1.0/1.0	76.7 (23.2)	0.6/0.6
Role functioning	51.8 (28.8)	51.9 (29.1)	82.4 (25.9)	1.2/1.1	70.5 (32.8)	0.6/0.6
Emotional functioning	61.6 (25.5)	62.1 (25.9)	79.2 (25.1)	0.7/0.7	71.4 (24.2)	0.4/0.4
Cognitive functioning	52.5 (28.8)	54.5 (29.0)	83.7 (22.6)	1.4/1.2	82.6 (21.9)	1.4/1.2
Social interference/social functioning^3^	54.6 (31.4)	54.6 (31.4)	86.5 (24.2)	1.2/1.2	75.0 (29.1)	0.7/0.7
Fatigue	58.3 (27.0)	58.3 (27.0)	29.9 (26.7)	1.1/1.1	34.6 (27.8)	0.9/0.9
Pain	44.9 (30.8)	44.9 (30.8)	23.4 (26.5)	0.8/0.8	27.0 (29.9)	0.6/0.6
Sleep problems/insomnia^4^	48.9 (28.3)	47.5 (35.4)	28.5 (31.2)	0.6//0.6	28.9 (31.9)	0.6/0.6
Financial difficulties	27.2 (34.6)	27.2 (34.6)	12.2 (26.2)	0.5/0.5	16.3 (28.1)	0.4/0.4

LEC, Late Effects Clinics.

1 General population normative data for the EORTC QLQ-C30 health-related quality of life [17].

2 EORTC QLQ-C30 Reference Values, July 2008 [18].

3 Social interference: social functioning in QLQ-C30.

4 Sleep problems: insomnia in QLQ-C30.

There was no significant difference in the Global Health scores between women and men in our data, which was also the case for Physical functioning, Role functioning, Emotional functioning, and Social interference. However, women reported a significantly lower Cognitive functioning score (*p* = 0.007) compared to men, with a mean difference of 11.4, which is considered medium clinically relevant by Cocks et al. [21]. Women also had a worse fatigue score (*p* = 0.017) with a mean difference of 9.5 (small clinically relevant). Concerning age, there was no significant difference in all scales between younger (≤ 39 of age) and older patients (> 39). The scales/items that are not comparable with the scales/items from EORTC QLQ-C30 appear to be in alignment with the other scales (Table 3).

**Table 3:** Overview of the scales from the EORTC SURV100 questionnaire that are not comparable with previous scales or norm data/EORTC reference values, including number of responses.

Scales (number of responses)	QOL-SURV100 Danish data (Odense) LEC baseline Mean (SD)
Body image (234)	53.5 (30.1)
Symptom awareness (234)	50.0 (27.8)
Health distress (235)	51.7 (29.9)
Loss of income (156)	47.2 (43.6)
Negative health outlook (235)	51.1 (24.7)
Problems insurances, loans, mortgages (105)	22.2 (34.5)
Social isolation (234)	35.8 (31.1)
Symptom checklist (236)	36.1 (19.0)
Work (133)	53.6 (30.6)
**Optional Scales**	
Deeper meaning (217)	26.9 (31.4)
Fertility (33)	46.5 (42.4)
Partner relation stronger (170)	58.6 (36.7)
Positive life outlook (232)	50.1 (25.4)
Positive health behavior change (233)	50.0 (26.0)
Positive impact on behavior toward others (222)	48.2 (28.2)
Positive social functioning (230)	53.5 (29.1)
Sexual functioning (220)	27.4 (24.6)
Sexual pleasure (102)	53.6 (32.9)
Sexual problems (220)	42.7 (37.6)
Sexual problems female (78)	45.3 (44.0)
Sexual problems when sexually active (105)	34.1 (35.0)
Sexual problems male (53)	48.4 (44.1)
Treated differently (231)	23.1 (27.4)
Worry cancer risk family (234)	36.5 (34.9)
Worry impact of cancer on children (192)	58.5 (33.2)

LEC: Late Effects Clinics.

## Discussion

The current study found that patients who have been referred to an LEC in Denmark have a significantly lower HRQoL on all scales than the general Danish population and patients who have just been diagnosed with cancer. These differences are considered to be meaningful and have practical significance for all the scales. The findings suggest that the regional aim of helping patients suffering from late effects gain a better QoL is much needed.

We observed an overrepresentation of women referred to the clinic and found breast cancer to be the most common cancer diagnosis. The overall number of Danish women living with cancer is higher (55%) than that of men (45%) [22]. However, almost three out of four patients referred to the LEC were women. Most of the women were referred by their general practitioner and often at their own initiative. This is in line with the general trend where women use more health care services, including cancer rehabilitation, than men [23–25]. It could simply be a reflection of diagnosis as the number of patients with a breast cancer diagnosis referred to the LEC (40%) does not mirror the Danish breast cancer survivor prevalence of 20% [22]. Many patients diagnosed with breast cancer have received extensive and long-term treatment with curative intent, often including surgery, chemotherapy, radiotherapy, and adjuvant endocrine treatment. Hence, they become long-term survivors with a high risk of late effects [26].

Thirty-six per cent of Danes have a higher education [27], which does not correspond to the 60% of the patients included in the study, indicating that even though these patients are challenged by multiple late effects and have a low HRQoL, they are still more likely to find resources to seek help and initiate a referral to an LEC. This may give rise to concerns if there is a larger number of people with late effects who do not find their way to support and warrant a more systematic approach to identify persons with late effects.

Cohabiting status may also be an issue when discussing referrals to an LEC. According to Statistics Denmark, approximately 40% of Danes live without a partner [28], whereas only 27% of the patients seen in the LEC reported living alone. The median age of Danish cancer survivors is 67 [29], which is 10 years older than the median age of the patients seen in our clinic (57 years), which may affect cohabiting status. Moreover, studies show that married or cohabiting people try to alleviate each other’s mental health concerns, for example, by encouraging their partner to seek help [30], and that breast cancer patients who are married have a better prognosis than unmarried patients [31], suggesting that a supporting spouse may have a positive effect on a partner’s health-seeking behavior. Thus, patients may be motivated by their partner to seek help either as an act of caring or because the patients’ late effects may influence family life negatively.

According to the WHO, a citizen’s health literacy is a key factor impacting social inequality and health. A review on the role of health literacy in cancer care from 2021 also concludes that patients with greater understanding and knowledge of their disease and rights may take a more active role in their own care and be better at navigating the system, resulting in poorer participation for the patients who experience difficulties with health literacy [32]. This may also be relevant for our clinic, resulting in an underrepresentation of patients with low health literacy. A recent cross-sectional study among more than 27,000 Danish cancer survivors also concludes that survivors with a short education are at greater risk of impaired HRQoL compared with those who have a long education. This underlines the need for an increased focus on these patients in cancer survivorship [33] and should be focal point for future collaboration between LECs and referring healthcare professionals.

We did not find any significant difference in the overall HRQoL between younger and older patients, which may be due to the low number of young people in our cohort. Only 10% (*n* = 24) of the patients were below the age of 40 years. We detected some significant differences between the sexes. Women scored significantly lower on the cognitive functioning scale than men, and their Fatigue score was significantly higher. This may be explained by the fact that women often have multiple roles, that is household keepers, family managers, wives, mothers, employees, and friends [34]. Before their cancer diagnoses, these women may have been able to fulfill all roles, meeting own, and other’s. However, getting cancer may result in a reduced ability to fulfill these expectations, leading to feelings of guilt and high levels of stress [35], which may challenge their cognitive capacity, result in sleep problems, and impact their levels of fatigue. Factors, which again, may influence their HRQoL negatively and their ability to work [26, 36].

## Strength and limitations

It is an obvious strength that all patients seen in the clinic agreed to participate in the database and nearly all completed the questionnaire. The fact that patient reporting was used as a dialog tool in the clinical encounter may have played a positive role. Although SURV100 is a newly developed questionnaire and there are no norm data yet, it was still possible to compare some of our findings with norm data from the QLQ-C30 questionnaire. Moreover, after we have collected follow-up data (6, 12, and 24 months), we will be able to follow changes over time in the scales for which there were no norm data. These results, including changes over time, will be published at a later time point. Furthermore, our study can provide a basis for comparison for researchers and clinicians who intend to use SURV100 in the future. It is a limitation that the majority of the patients had a higher education, excluding patients with low health literacy. Moreover, the patients may be so eager to get help that they unconsciously score worse to better their chances of being heard and having their late effects relieved, which is a potential bias.

Further research is needed to elucidate how these multiple late effects interact interconcurrently and negatively affect QoL and to compare our patient population to Danish cancer survivors in general.

### Perspectives

The number of cancer patients with late effects will continue to increase as more Danes are diagnosed with cancer and treatments improve. Accordingly, awareness among healthcare professionals on late effects should be increased during the cancer trajectory [11]. Prevention should focus on providing patients with information on possible late effects. – thus if patients are better prepared, they may also be better able to cope in the long run. Johansen et al. suggest that the cornerstone of future survivorship care should be patient education and follow-up care prompted by the patients themselves, for example, through PRO reporting [37]. The QLQ-SURV100 could be a relevant tool for this purpose. Utilizing PRO for screening and monitoring during survivorship could facilitate the early detection of late effects, preventing their progression and preserving patients’ HRQoL. However, educating patients and their family caregivers and monitoring patient reporting in-between and at follow-up visits may require additional time, which may not be possible in a strained oncology healthcare system where the pressure to detect, diagnose, and treat cancer within specific time limits is immense [38].

## Conclusion

Patients who have been referred to an LEC in Denmark have a significantly lower HRQoL than the general Danish population and patients who have just been diagnosed with cancer, suggesting that the regional aim of helping patients suffering from late effects gain a better QoL is in dire need. Aiding the most challenged patients in LECs may help the patients and their families cope with their new life situation, resulting in an improved HRQoL. Increased awareness among healthcare professionals on late effects, including better patient psychoeducation during the cancer trajectory, may be the way forward to detect and manage late effects at an earlier time point and remedy the consequences.

## Data Availability

The datasets generated during and/or analyzed during the current study are available from the corresponding author upon reasonable request.
